# Intracranial invasion of a mast cell tumour in a dog: A case report and review of the literature

**DOI:** 10.1002/vms3.1402

**Published:** 2024-03-07

**Authors:** Edward Kingsbury, Petros Odatzoglou, A. L. Peschard, Hannah Wong, Richard Elders

**Affiliations:** ^1^ Department of Veterinary Medicine University of Cambridge Cambridge Cambridgeshire UK

**Keywords:** dogs, intracranial, mast cell tumour, metastatic, osteolysis

## Abstract

An 11‐year‐old, female‐neutered beagle was presented with a growing soft tissue mass arising within the deep tissues of the left cranial cervical region. At presentation, facial asymmetry was evident along with palpable lymphadenomegaly. Magnetic resonance imaging demonstrated a locally invasive cervical mass with intracranial invasion through focal osteolysis of the occipital bone. After antihistamine administration, cytology confirmed mast cell tumour (MCT) with metastasis to local lymph nodes and liver. The owner chose to pursue lomustine and prednisolone, which were dispensed, but, before home administration, prolonged seizures/status epilepticus occurred prompting euthanasia. Postmortem examination confirmed a high‐grade MCT associated with, and infiltrating through, muscle, calvarium, dura mata, leptomeninges and the underlying brain. We present the clinical, imaging, and pathological findings of an unprecedented case of extracranial MCT tumour causing osteolysis of an imperforate flat bone (occipital bone) and intracranial invasion.

## Graphical abstract

This case report documents the clinical presentation, imaging findings and pathology in a unique case of a confirmed extracranial mast cell tumour causing focal osteolysis and intracranial invasion in a dog. To our knowledge intracranial invasion or any osteolysis have yet to be described as biological behaviour of canine MCTs.

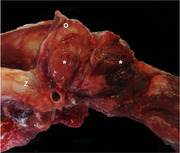

## 1 INTRODUCTION

Canine mast cell tumours (MCTs) are the most common integumentary malignancy in dogs, accounting for 16%–21% of all skin tumours (Bostock, 1986). They have a variable biological behaviour, ranging from nondisseminating solitary masses to widespread metastasis and, occasionally, systemic disease (Garrett, 2014, Kiupel & Camus, 2019). The majority of subcutaneous MCTs have relatively better outcomes compared to cutaneous MCTs, with longer survival time and extended time to disease progression, although recent studies have highlighted that subcutaneous tumours can be associated with a higher nodal metastatic rate at presentation (Ferrari et al., 2021; Lapsley et al., 2021; Marconato et al., 2023; Pecceu et al., 2020). Primary visceral or leukaemic presentations are rare (Takahashi et al., 2000). Location has been associated with biological behaviour, with more aggressive MCTs noted on the mucosae/mucocutaneous junctions, perioral and inguinal regions (Cahalane, 2004; Gieger et al., 2003; Sfiligoi et al., 2006). Histopathological features of the neoplastic mast cells are used for prognostication and therapeutic decision making (Sledge et al., 2016). Grading systems focus on distinguishing poorly differentiated tumours from better differentiated tumours, with the Patnaik and Kiupel schemes being most widely used (Kiupel & Camus, 2019; Kiupel et al., 2011; Patnaik et al., 1984; Willmann et al., 2021). Although visceral dissemination is associated with a shorter prognosis, there is less consensus on the prognostic impact of, and therapy indicated for, lymph node metastases. Proposed nodal metastatic status classifications systems are inconsistently applied in recent research studies (Krick et al., 2009; Stefanello et al., 2009; Weishaar et al., 2014). Other features including Ki‐67 and c‐KIT IHC, *c‐kit* sequencing, and AgNOR staining have been used to aid prognostication (Kiupel & Camus, 2019).

Canine mast cell histiogenesis and mast cell tumour aetiology have not yet been fully elucidated, but carcinogenesis is considered multifactorial with a genetic component (Welle et al., 2008). Although clinical features and disease progression of typical MCTs are well described, less common presentations evade prompt diagnosis and treatment, perhaps limiting efficacy. Bone involvement is rarely reported in the veterinary MCT literature. One published case of a disseminated MCT, lacking a detectable primary tumour, involved the medullary cavities of the sphenoid bones; however, there was no evidence of osteolysis (Beltran et al., 2010). In a case series of four dogs with intranasal MCTs, one dog had intracranial extension of the tumour through the cribriform plate and, subsequently, developed seizures thought, but not proven, to be a sequela of that invasion (Khoo et al., 2017). Another case report described an extra‐axial intracranial MCT of a dog with rostral extension into the nasal cavity, but this extended through the porous cribriform plate, rather than exhibiting definitive bone destruction (Yang et al., 2022). In the human medical literature, a case report described a localised intracranial mast cell tumour that was also reported to have originated in the cranium (Guenther et al., 2001). To the authors’ knowledge, no previous reports have described overt osteolytic activity resulting in intracranial invasion as part of MCT pathogenesis.

This case report documents the complete clinical, imaging, clinicopathological, and necropsy findings in a unique case of a confirmed extracranial MCT causing focal osteolysis and intracranial invasion.

## 2 CASE DESCRIPTION

An 11‐year‐old female‐neutered beagle was presented to the Medical and Radiation Oncology service at the Queen’s Veterinary School Hospital for assessment of a rapidly growing mass within the deep soft tissues of the left side of the cranial neck and ear base. The mass was previously cytologically diagnosed as a MCT by the referring veterinary surgeon using rapid in‐clinic stains and had decreased in size following medical therapy with chlorphenamine (Piriton, GSK, UK) (4 mg, p/o, q8h) and robenacoxib (Onsior, Elanco, UK) (20 mg, p/o, q24h). At admission, facial asymmetry was evident, with the tissues over the temporal bones on the left protruding laterally compared with the right. Palpation of the mass revealed a firm, well circumscribed, nonpainful, nonmobile, deep‐seated mass measuring approximately 6.5 × 8 × 4 cm, extending rostrally from the caudal aspect of the left ear base and dorsally to the left parietal region. The left submandibular lymph node was enlarged in size. Clinical examination was otherwise unremarkable. Throughout the physical examination, the patient was behaviourally and neurologically appropriate, with no spontaneous or inducible neck pain and no overt neurological deficits.

Complete blood count and serum biochemistry analyses were wholly unremarkable. Inflated thoracic radiography, cervical magnetic resonance imaging (MRI), abdominal ultrasonography and fine needle aspirates were performed under general anaesthesia. The images were interpreted by a European College of Veterinary Diagnostic Imaging (ECVDI) approved small animal track resident (A.‐L. P.) under the supervision of an ECVDI Diplomate.

The MRI revealed a large multilobulated mass extending from the caudal margin of the left external ear canal rostrally to the level of the C4 vertebra caudally. The mass was invading, rather than displacing, the left temporalis, semispinalis capitis and mastoid part of the cleidocephalicus and sternocephalicus muscles. The mass had a heterogeneous hyperintense signal on T2‐weighted (T2W) sequence, marked hyperintensity on the dorsal Short Tau Inversion Recovery (STIR) sequence, mild hyperintensity compared with muscle on T1‐weighted (T1W) sequence, with marked contrast enhancement following intravenous administration of gadolinium (Gadovist^TM^, Bayer plc, UK). The mass was associated with focal lysis of the underlying occipital bone, and extended through this skull defect, forming a broad‐based intracranial extra‐axial nodule. The latter was associated with thickening and contrast enhancement of the adjacent meninges, a ‘dural tail’. This extra‐axial nodule impinged on the left cerebellar hemisphere, causing right sided deviation of the cerebellum, and was associated with a focal rounded T2W markedly hyperintense, fluid attenuated inversion recovery (FLAIR)‐suppressing, T1W hypointense, and noncontrast enhancing fluid collection.

A second, smaller broad‐based, peripheral, extra‐axial nodular lesion of similar signal characteristics to the primary mass (above) was visible superficial to the tentorium in the most caudoventral aspect of the left occipital lobe. Thickening and contrast enhancement of the adjacent meninges were also associated with this mass. The lateral and third ventricles of the brain were bilaterally enlarged, and there was diffuse loss of the normal gyral pattern of the forebrain. The cranial cervical cord showed an extensive, well‐defined cylindrical region of intramedullary T2W hyperintensity, extending dorsally from the central canal. This was hypointense on T1W sequences, only centrally suppressed on FLAIR and showing no contrast enhancement. Moderate rounding and enlargement (0.85 cm) of the left retropharyngeal lymph node was noted compared with the right (0.46 cm).

Staging with thoracic radiography showed no overt evidence of dissemination. Abdominal ultrasonography revealed multifocal small rounded hypoechoic nodules in the liver and the spleen was diffusely enlarged with a coarse heterogeneous echogenicity. Ultrasound guided fine needle aspirates from the left and right medial retropharyngeal and mandibular lymph nodes, spleen, and liver were examined by a board‐certified clinical pathologist. Cytological examination confirmed certain mast cell metastasis to the left medial retropharyngeal lymph node and liver, and possible metastasis to the right medial retropharyngeal lymph node and spleen (Stefanello et al., 2009). The mandibular lymph nodes showed mild lymphoid reactivity and no evidence of metastasis.

The discordance between the imaging findings and the original cytological diagnosis prompted ultrasound guided cytological sampling of the peripheral cervical portion of the primary mass to refute a differential diagnosis of a mast cell tumour overlying an unrelated infiltrative mass. Assessment by a board‐certified clinical pathologist confirmed the original diagnosis of a mast cell tumour. Given the MRI findings, a cisterna magna cerebrospinal fluid (CSF) aspirate was attempted but was unsuccessful, thought likely due to the disrupted anatomy of the area secondary to the presence of the cervical mass.

A treatment plan with high dose chemotherapy using lomustine (CCNU, Chemopet, UK) (70–75, mg/m^2^, q21d) and prednisolone (Prednidale, Dechra, UK) (1.3 mg/kg, p/o, q24) was made after discussion of several therapeutic options with the owners. The patient was discharged with instructions to start Omeprazole (Omeprazole, Teva, UK) (10 mg, p/o, q12h), to continue chlorphenamine as above, and to discontinue robenacoxib to facilitate a wash‐out period before prednisolone administration. Chemotherapy, however, was never initiated, as the patient developed overt epileptic seizure activity within 18 h of discharge, progressing to status epilepticus, and was ultimately euthanised at the referring practice within 24 h of discharge.

## 3 PATHOLOGY

**FIGURE 1:**
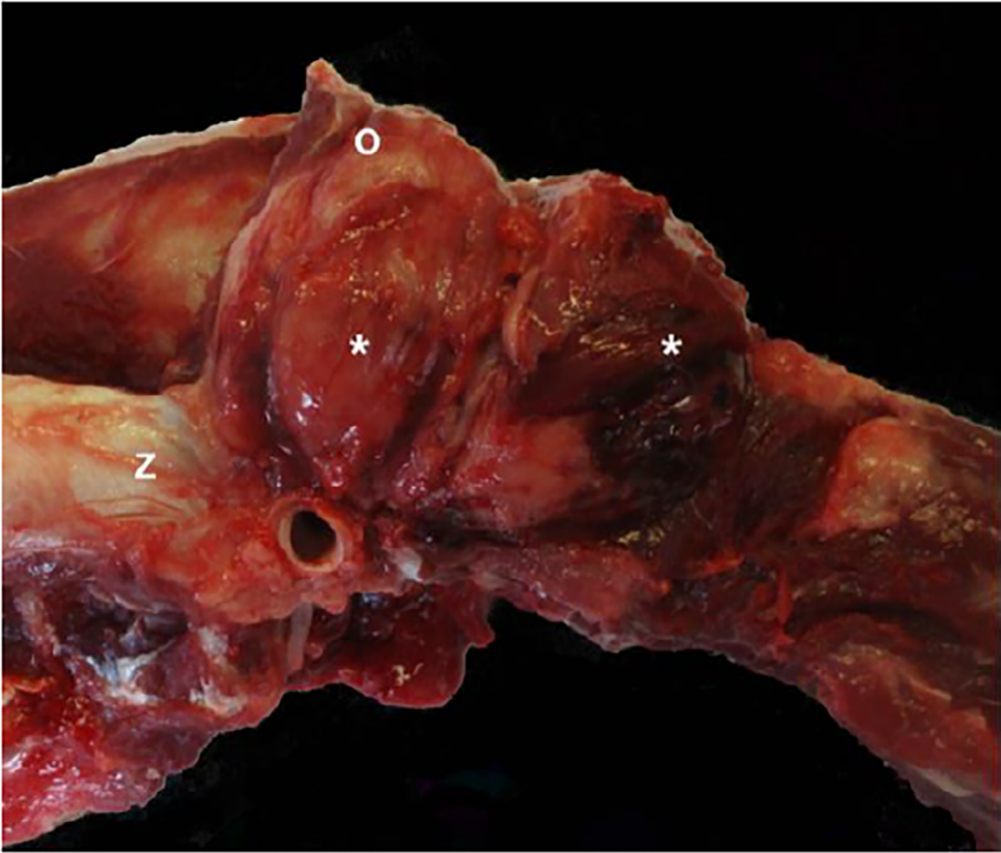
Canine, head and cranial neck. The muscles of the cranio‐laterodorsal neck are expanded by a multilobulated cream‐red soft tissue mass (*), which is bordered cranially by the occipital bone (O) and ventrally by the external ear canal and base of the zygomatic process (Z).

**FIGURE 2:**
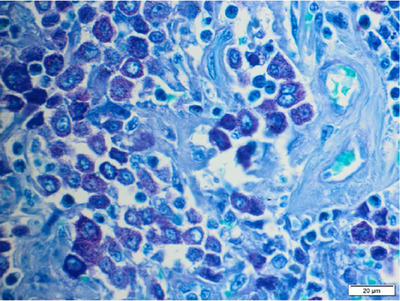
Canine, deep cervical mass, toluidine blue ×600 magnification. Neoplastic cells contain large numbers of metachromatic cytoplasmic granules, indicating mast cells lineage.

At postmortem examination, the lesions documented were consistent in terms of dimensions and locations with the imaging findings, with no additional changes such as an anaphylactoid process. The skeletal muscle and calvarium of the left occipital region immediately caudal to the horizontal ear canal were infiltrated by a 6×5×4 cm, multilobulated, cream‐to‐red mass composed of sheets of neoplastic, well‐granulated mast cells (Figure 1). Neoplastic cells displayed marked anisocytosis and anisokaryosis, 12 mitotic figures per 2.37 mm^2^, and were mixed with large numbers of eosinophils (Figure 2).

**FIGURE 3:**
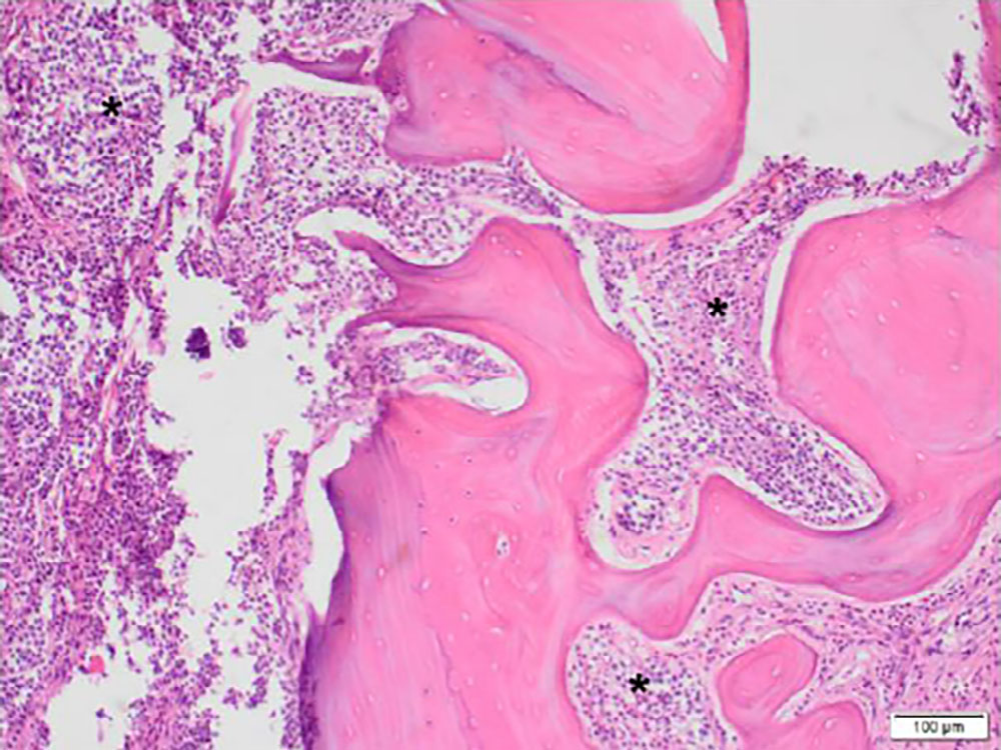
Canine, occipital bone, H&E ×100 magnification. Occipital bone invaded and replaced by sheets of neoplastic mast cells (*).

**FIGURE 4:**
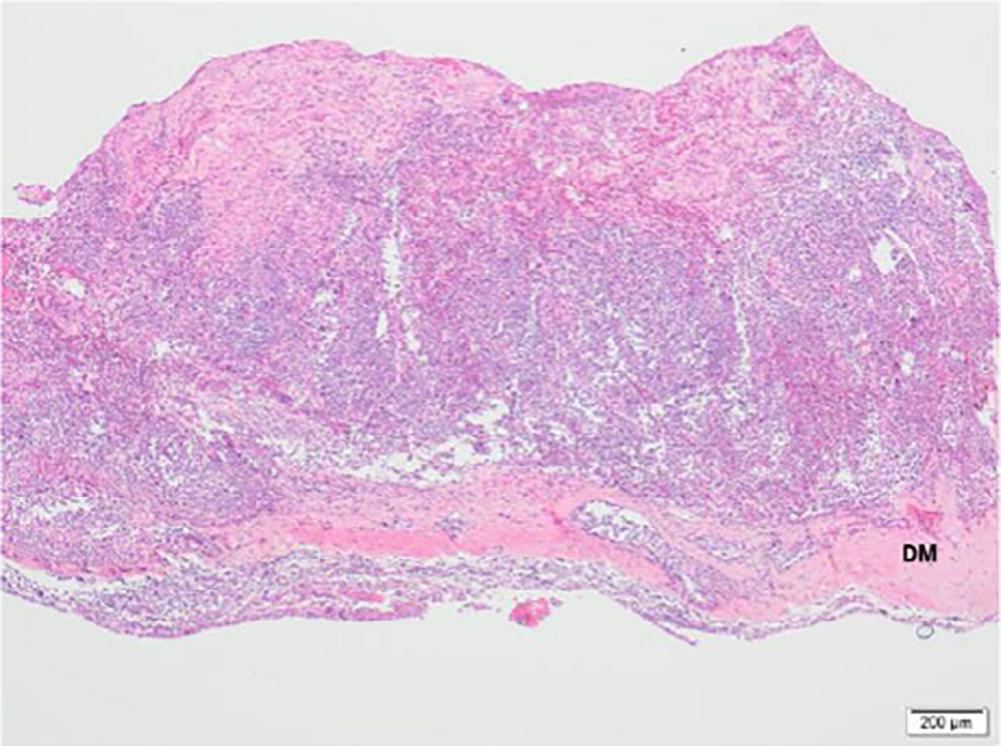
Canine, meninges, overlying occipital lobe left, H&E ×40 magnification. Expansion of the dura mata (DM), in the region of the occipital bone infiltration, by neoplastic mast cells mixed with mature fibrous tissue.

**FIGURE 5:**
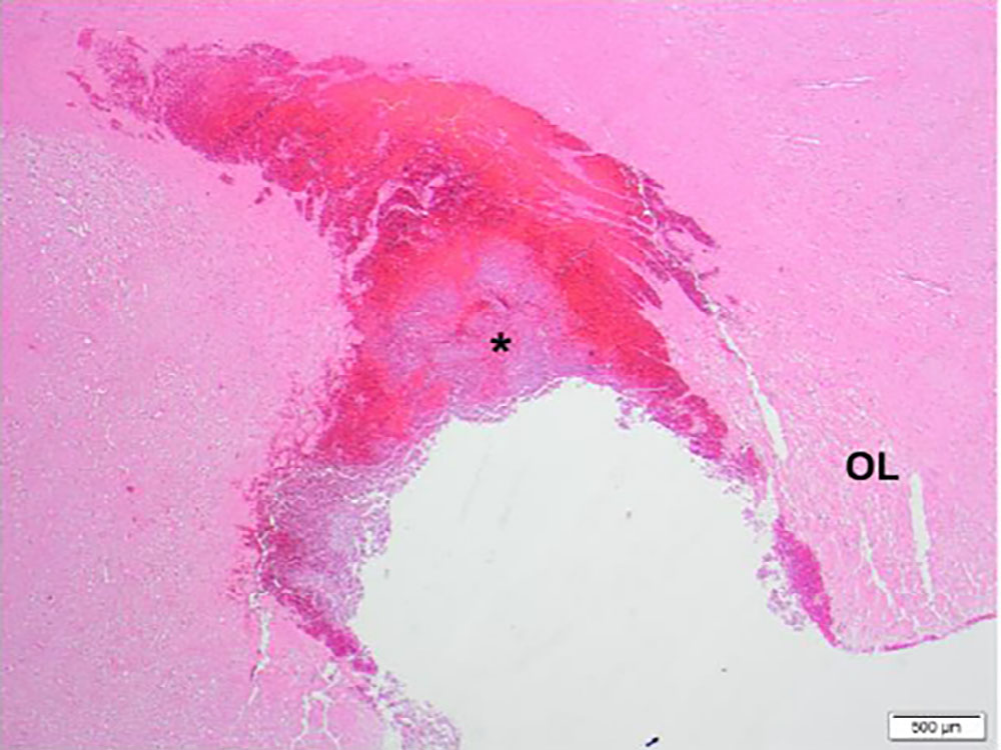
Canine, brain, cerebral cortex, caudal occipital lobe left (OL), H&E ×20 magnification. Infiltration and replacement of the neuroparenchyma by haemorrhage and neoplastic mast cells (*).

Focally, the neoplastic cells completely replaced the calvarium (Figure 3), and, in adjacent areas, neoplastic mast cells and eosinophils effaced hematopoietic cells within medullary spaces. The subjacent dura mata overlying the left occipital lobe was expanded by sheets of neoplastic mast cells (Figure 4) and was adherent to the leptomeninges. In the superficial cervical portion of the mass, around the area sampled for cytology, there was mild, localised, likely iatrogenic haemorrhage, which did not extend into deeper tissues or intracranially. On the caudoventral aspect of the left occipital lobe, there was a 1.5 × 1 × 0.5 cm focal area where grey and white matter was replaced by neoplastic mast cells and haemorrhage (Figure 5).

The left submandibular lymph node had overt mast cell metastasis, and the right submandibular lymph node was designated as a premetastatic lymph node (Weishaar et al., 2014). Microscopic examination of the spleen, liver and femoral bone marrow did not confirm histologically detectable mast cell metastasis.

The cervical spinal cord at mid C1 to caudal C2 was multifocally and irregularly brown in colour, with both the grey and white matter expanded by irregular areas of nonependymal lined clear space (syringomyelia).

## 4 DISCUSSION

The present case report describes a MCT associated with the deep cervical soft tissues in a dog showing metastatic and osteolytic behaviour, with invasion of the calvarium and underlying brain parenchyma. Poorly differentiated MCTs are locally invasive as well as metastatic, which, in the case of a dermal MCT, can result in local invasion of the subcutis and muscles. In this case, the MCT had no involvement of the overlying skin or subcutis, suggesting a potential soft tissue or bony origin. The distribution of the other neoplastic lesions was consistent with metastasis from the primary cervical mass. A diagnosis of disseminated mastocytosis was excluded due to the size of the tumour, local lymph node involvement with lack of lymphadenopathy and bone marrow involvement elsewhere in the body.

Bone invasion and osteolytic activity is rarely attributed to canine MCT. In a case series of canine spinal MCTs, three out of four MCTs were confined to the epidural space, two of which were high‐grade MCTs exhibiting no involvement nor periosteal reaction of the adjacent vertebrae. The other case was also high‐grade showing mild periosteal reaction of the adjacent rib head with no bone lysis observed on advanced imaging (Moore et al., 2017).

In contrast, several other tumour types arising in the cranial neck region are more commonly associated with bone invasion and osteolytic activity. A chondrosarcoma on the skull of a dog caused osteolysis to the occipital and parietal bones with expansion into the cranium; however, in contrast to the current case, there was no invasion into the brain parenchyma (Kim et al., 2007). Squamous cell carcinomas of humans and animals also cause frequent osteolysis (Steinmetz, 2017; Som et al., 1996).

The pathogenesis of tumour‐induced osteolysis remains unclear but might include an increase of osteoclastic activity with simultaneous suppression of osteoblastic activity via tumour‐bone microenvironment interactions. The RANK, RANKL, OPG, Notch and Wnt pathways, along with various chemokines and interleukins, have been shown to be involved in this process in cases of multiple myeloma (Terpos et al., 2018). Mast cells are reported to have an osteocatabolic effect, promoting osteoclast formation via release of histamine, interleukin‐6 and tumour necrosis factor while also inhibiting osteoblast activity. This process could have contributed to the marked tumour‐induced osteolysis observed in the current case (Biosse‐Duplan et al., 2009; Ragipoglu et al., 2020).

The biological behaviour of this MCT draws parallels with a rare form of mast cell neoplasia in humans, called mast cell sarcoma (MCS). The nomenclature of the most common human and canine mast cell neoplastic presentations are distinct, and terms are not synonymously used between species (Matsumoto et al., 2022). Mast cell sarcomas are rare, localised forms of mast cell neoplasia that lack systemic dissemination, with 34 cases reported currently in human literature (Matsumoto et al., 2022). Initially, MCS can be misinterpreted as soft tissue sarcomas, given several features they have in common, which include frequent bone involvement, a solitary primary site within deep soft tissues, often lacking subcutaneous/cutaneous involvement, lacking muscular invasion, and pleomorphic cell morphology (Yang et al., 2022). Interestingly, human MCS cases share many similar characteristics with this current canine case. Out of the 34 human MCS cases reported, 62% showed bone involvement (Matsumoto et al., 2022). Initially localised in soft tissues or bone, MCS can be extremely invasive into adjacent bony structures, including vertebrae, femora and sternebrae (He et al., 2017; Inaoui et al., 2003; Krauth et al., 2007). Many human MCS cases are classified as originating from within the bone itself. In the current case, there was close approximation of the primary mass to the skull, combined with a lack of dermal or subcutaneous involvement. It could be hypothesised, therefore, that the outer cortex's periosteum or occipital bone marrow might have been the site of initial mast cell transformation, with the bulk of the mass expanding into the deep neck tissues along the path of least resistance and a smaller presence intracranially.

Dissemination of MCS is possible and can resemble a mast cell leukaemia in the terminal stages, which is reported in around 30% of MCS cases (Monnier et al., 2016; Raymond et al., 2020). In the current canine case, blood film evaluation showed no evidence of mast cells in circulation, while histology of bone marrow from a remote location (other than in the occipital bone) showed no mast cell infiltrates. Disagreement between cytological and histopathological samples used for staging of a MCT is frequently observed (Ku et al., 2017; Lapsley et al., 2021). For example, many histopathologically evident lymph node metastases elude detection cytologically by fine needle aspiration (Fournier et al., 2018). The disagreement in the current case seems to have run in the counter direction, with cytologically suspected and definite dissemination to the liver and spleen, respectively, not confirmed on histopathology, possibly reflecting different areas of the viscera being sampled by the two methodologies.

**FIGURE 6:**
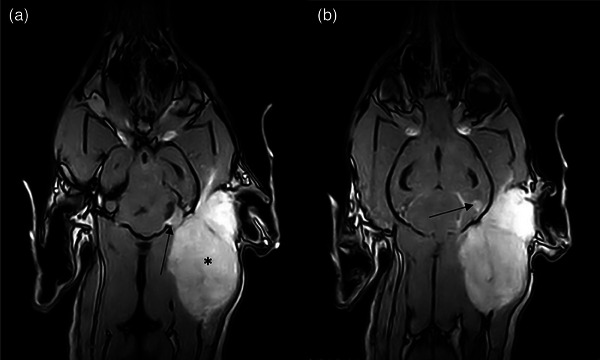
(a) Magnetic resonance imaging findings show the mass (*) is associated with focal lysis of the underlying occipital bone (arrow). The mass extends into the skull, forming a broad‐based intracranial extra‐axial nodule against the internal surface of the occipital bone. (b) Moving dorsally, a second, smaller broad‐based, peripheral, extra‐axial nodular lesion of similar signal characteristics to the main/primary mass (arrow) queried as a ‘drop metastases’. Notice how the occipital bone is intact in this area.

The second brain lesion, distinct from the calvarium infiltration observed on imaging and at postmortem examination, has features that are attributed to ‘drop metastases’, where a neoplasm disseminates via the CSF resulting in further downstream lesions across the central nervous system/CSF drainage pathway (Figure 6a and b) (Vigeral et al., 2018). In this case, the mass is primarily deep cervical in location, and it has been hypothesised that it is periosteal/marrow in origin, therefore making it extra‐axial. It is noted that some clinicians reserve the term ‘drop metastases’ specifically for metastasis of primary intra‐axial neoplasms.

In the medical literature, drop metastases affect approximately 0.1%–0.4% of all cancer patients (Kalaycı et al., 2004; Lee et al., 2007) and mostly involves CNS primary tumours, including glioblastoma multiforme, meningioma, and pineal tumours (Solomou, 2017). It is also described as a rare complication of tumours metastatic to the CNS, including nonsmall cell lung cancer, lymphoma and melanoma (Dam‐Hieu et al., 2009). The rise of advanced diagnostic imaging with MRI has increased the sensitivity for detecting drop metastasis in living patients (Dam‐Hieu et al., 2009). For the current case, other differentials for the possible drop metastasis included local extension of the neoplastic mast cells in the meninges, compressed blood vessels resulting in an infarction, and loss of brain tissue, and haemorrhage.

A limitation of the current case is the lack of a full neurological/diplomate neurologist examination at presentation. Although no obvious neurological deficits were evident at presentation to prompt such a request, including the dog pulling strongly on the lead, an examination by a diplomate neurologist might have revealed subtle neurological changes, hinting at brain invasion before imaging. Use of CT rather than MRI would not be expected to alter the case outcome due to the nonresectable nature of the lesion being clearly identifiable using MRI as the imaging modality.

The current case highlights the challenge that mast cell neoplasia continues to pose in practice, given their unpredictable biological behaviour, metastatic potential and variable invasiveness. Based on this case description, MCT should be considered a rare differential for deep‐tissue masses displaying osteolytic activity on diagnostic imaging.

## AUTHOR CONTRIBUTIONS

E. Kingsbury: conceptualisation, investigation, writing – original draft, writing – review & editing. P. Odatzoglou: investigation, writing – review & editing. L. Peschard: investigation, writing – review & editing. H. Wong: conceptualisation, investigation, software, supervision, writing – review & editing. R. Elders: conceptualisation, investigation, resources, supervision, writing – review & editing.

## FUNDING

The authors did not receive any specific funding for this work.

## CONFLICT OF INTEREST STATEMENT

The authors have declared that no competing interests exist.

## ETHICS STATEMENT

The authors do not perceive any ethical impediments whatsoever as the case presents no original research data.

### PEER REVIEW

The peer review history for this article is available at https://publons.com/publon/10.1002/vms3.1402.

## Data Availability

Data sharing is not applicable to this article as no new data were created or analysed in this study.

## References

[vms31402-bib-0001] Beltran, E. , de Stefani, A. , Stewart, J. , Risio, L. D. , & Johnson, V. (2010). Disseminated mast cell tumor infiltrating the sphenoid bone and causing. Veterinary Ophthalmology, 13(3), 184–189. 20500719 10.1111/j.1463-5224.2010.00776.x

[vms31402-bib-0002] Khoo, A. , Lane, A. , & Wyatt, K. (2017). Intranasal mast cell tumor in the dog – A case series. Canadian Veterinary Journal, 58(8), 851–854. PMC5508967 28761193

[vms31402-bib-0003] Biosse‐Duplan, M. , Baroukh, B. , Dy, M. , De Vernejoul, M.‐C. , & Saffar, J.‐L. (2009). Histamine promotes osteoclastogenesis through the differential expression of histamine receptors on osteoclasts and osteoblasts. American Journal of Pathology, 174, 1426–1434. 19264900 10.2353/ajpath.2009.080871 PMC2671373

[vms31402-bib-0004] Bostock, D. E. (1986). Neoplasms of the skin and subcutaneous tissues in dogs and cats. British Veterinary Journal, 142, 1–19. 3947927 10.1016/0007-1935(86)90002-3

[vms31402-bib-0005] Inaoui, R. , Petit, B. , Jaccard, A. , Bertin, P. , & Trèves, R. (2003). Aggressive systemic mastocytosis. Joint Bone Spine, 70, 64–66. 12639621 10.1016/s1297-319x(02)00015-5

[vms31402-bib-0006] Cahalane, A. K. P. S. , Barber, L. G. , Duda, L. E. , Henry, C. J. , Mauldin, G. E. , Frimberger, A. E. , Cotter, S. M. , & Moore, A. S. (2004). Prognostic factors for survival of dogs with inguinal and perineal mast cell tumors treated surgically with or without adjunctive treatment: 68 cases (1994–2002). Journal of the American Veterinary Medical Association, 225(3), 401–408. 15328716 10.2460/javma.2004.225.401

[vms31402-bib-0007] Dam‐Hieu, P. , Seizeur, R. , Mineo, J.‐F. , Metges, J.‐P. , Meriot, P. , & Simon, H. (2009). Retrospective study of 19 patients with intramedullary spinal cord metastasis. Clinical Neurology and Neurosurgery, 111, 10–17. 18930587 10.1016/j.clineuro.2008.06.019

[vms31402-bib-0008] Ferrari, R. , Boracchi, P. , Chiti, L. E. , Manfredi, M. , Giudice, C. , De Zani, D. , Spediacci, C. , Recordati, C. , Grieco, V. , Gariboldi, E. M. , & Stefanello, D. (2021). Assessing the risk of nodal metastases in canine integumentary mast cell tumors: Is sentinel lymph node biopsy always necessary? Animals (Basel), 11, 2373. 34438830 10.3390/ani11082373 PMC8388797

[vms31402-bib-0009] Fournier, Q. , Cazzini, P. , Bavcar, S. , Pecceu, E. , Ballber, C. , & Elders, R. (2018). Investigation of the utility of lymph node fine‐needle aspiration cytology for the staging of malignant solid tumors in dogs. Veterinary Clinical Pathology, 47, 489–500. 30011068 10.1111/vcp.12636

[vms31402-bib-0010] Garrett, L. D. (2014). Canine mast cell tumors: Diagnosis, treatment, and prognosis. Veterinary Medicine (Auckl), 5, 49–58. 10.2147/VMRR.S41005 PMC7337164 32670846

[vms31402-bib-0011] Gieger, T. L. T. A. , Werner, J. A. , McEntee, M. C. , Rassnick, K. M. , & DeCock, H. E. (2003). Biologic behavior and prognostic factors for mast cell tumors of the canine muzzle: 24 cases (1990‐2001). Journal of Veterinary Internal Medicine, 17(5), 687–692. 14529136 10.1111/j.1939-1676.2003.tb02501.x

[vms31402-bib-0012] He, F. , Horny, H.‐P. , Boone, J. , Raza, A. , Griffith, M. , Hurley, P. , Dolan, M. , Cayci, Z. , Linden, M. A. , Mckenna, R. , & Ustun, C. (2017). Anaplastic mast cell sarcoma: A unique pathologic entity in mastocytosis. Leukemia & Lymphoma, 58, 1515–1517. 27808598 10.1080/10428194.2016.1250265

[vms31402-bib-0013] Kalaycı, M. , Çağavi, F. , Gül, S. , Yenidünya, S. , & Açıkgöz, B. (2004). Intramedullary spinal cord metastases: Diagnosis and treatment – An illustrated review. Acta Neurochirurgica, 146, 1347–1354. 15526223 10.1007/s00701-004-0386-1

[vms31402-bib-0014] Kiupel, M. , & Camus, M. (2019). Diagnosis and prognosis of canine cutaneous mast cell tumors. The Veterinary Clinics of North America. Small Animal Practice, 49, 819–836. 31178200 10.1016/j.cvsm.2019.04.002

[vms31402-bib-0015] Kiupel, M. , Webster, J. D. , Bailey, K. L. , Best, S. , Delay, J. , Detrisac, C. J. , Fitzgerald, S. D. , Gamble, D. , Ginn, P. E. , Goldschmidt, M. H. , Hendrick, M. J. , Howerth, E. W. , Janovitz, E. B. , Langohr, I. , Lenz, S. D. , Lipscomb, T. P. , Miller, M. A. , Misdorp, W. , Moroff, S. , …, Miller, R. (2011). Proposal of a 2‐tier histologic grading system for canine cutaneous mast cell tumors to more accurately predict biological behavior. Veterinary Pathology, 48, 147–155. 21062911 10.1177/0300985810386469 PMC8369849

[vms31402-bib-0016] Krauth, M. T. , Fodinger, M. , Rebuzzi, L. , Greul, R. , Chott, A. , & Valent, P. (2007). Aggressive systemic mastocytosis with sarcoma‐like growth in the skeleton, leukemic progression, and partial loss of mast cell differentiation antigens. Haematologica, 92, e126–129. 18055976 10.3324/haematol.11996

[vms31402-bib-0017] Krick, E. L. , Billings, A. P. , Shofer, F. S. , & Watanabe, S. , Sorenmo, K. U. (2009). Cytological lymph node evaluation in dogs with mast cell tumours: association with grade and survival. Veterinary and Comparative Oncology, 7, 130–138. 19453367 10.1111/j.1476-5829.2009.00185.x

[vms31402-bib-0018] Ku, C.‐K. , Kass, P. H. , & Christopher, M. M. (2017). Cytologic‐histologic concordance in the diagnosis of neoplasia in canine and feline lymph nodes: A retrospective study of 367 cases. Veterinary and Comparative Oncology, 15, 1206–1217. 27523399 10.1111/vco.12256

[vms31402-bib-0019] Lapsley, J. , Hayes, G. M. , Janvier, V. , Newman, A. W. , Peters‐Kennedy, J. , Balkman, C. , Sumner, J. P. , & Johnson, P. (2021). Influence of locoregional lymph node aspiration cytology vs sentinel lymph node mapping and biopsy on disease stage assignment in dogs with integumentary mast cell tumors. Veterinary Surgery, 50, 133–141. 33169849 10.1111/vsu.13537

[vms31402-bib-0020] Lee, S. S. , Kim, M. K. , Sym, S. J. , Kim, S. W. , Kim, W. K. , Kim, S.‐B. , & Ahn, J.‐H. (2007). Intramedullary spinal cord metastases: A single‐institution experience. Journal of Neuro‐Oncology, 84, 85–89. 17310265 10.1007/s11060-007-9345-z

[vms31402-bib-0021] Marconato, L. , Stefanello, D. , Solari Basano, F. , Faroni, E. , Dacasto, M. , Giantin, M. , Bettini, G. , Aresu, L. , Bonfanti, U. , Bertazzolo, W. , Annoni, M. , Lecchi, C. , & Sabattini, S. (2023). Subcutaneous mast cell tumours: A prospective multi‐institutional clinicopathological and prognostic study of 43 dogs. The Veterinary Record, 193, e2991. 37224084 10.1002/vetr.2991

[vms31402-bib-0022] Matsumoto, N. P. , Yuan, J. , Wang, J. , Shen, Q. , Chen, X. , Kim, Y. , Zuppan, C. W. , Chang, C.‐C. , Cui, W. , Chen, D. , Shi, M. , Gisriel, S. D. , Chen, M. , Xu, M. L. , & Pan, Z. (2022). Mast cell sarcoma: Clinicopathologic and molecular analysis of 10 new cases and review of literature. Modern Pathology, 35, 865–874. 35105959 10.1038/s41379-022-01014-w

[vms31402-bib-0023] Kim, H. , Nakaichi, M. , Itamoto, K. , & Taura, Y. (2007). Primary chondrosarcoma in the skull of a dog. Journal of Veterinary Science, 8(1), 99–101. 17322781 10.4142/jvs.2007.8.1.99 PMC2872705

[vms31402-bib-0024] Moore, T. W. , Bentley, R. T. , Moore, S. A. , Provencher, M. , Warry, E. E. , Kohnken, R. , & Heng, H. G. (2017). Spinal mast cell tumors in dogs: Imaging features and clinical outcome of four cases. Veterinary Radiology & Ultrasound: The Official Journal of the American College of Veterinary Radiology and the International Veterinary Radiology Association, 58, 44–52. 27723239 10.1111/vru.12429

[vms31402-bib-0025] Patnaik, A. K. , Ehler, W. J. , & Macewen, E. G. (1984). Canine cutaneous mast cell tumor‐ morphologic grading and survival time in 83 dogs. Veterinary Pathology, 21(5), 469–474. 10.1177/030098588402100503 6435301

[vms31402-bib-0026] Pecceu, E. , Serra Varela, J. C. , Handel, I. , Piccinelli, C. , Milne, E. , & Lawrence, J. (2020). Ultrasound is a poor predictor of early or overt liver or spleen metastasis in dogs with high‐risk mast cell tumours. Veterinary and Comparative Oncology, 18, 389–401. 31863546 10.1111/vco.12563

[vms31402-bib-0027] Som, P. M. , Silvers, A. R. , Catalano, P. J. , Brandwein, M. , & Khorsandi, A. S. (1996). Adenosquamous carcinoma of the facial bones, skull base, and calvaria – CT and MR manifestations. American Journal of Neuroradiology, 18(1), 173–175. PMC8337882 9010537

[vms31402-bib-0028] Guenther, P. P. , Huebner, A. , Sobottka, S. B. , Neumeister, V. , Weissbach, G. , Todt, H. , & Parwaresch, R. (2001). Temporary response of localized intracranial mast cell sarcoma to combination chemotherapy. Journal of Pediatric Hematology/Oncology, 23(2), 134–138. 11216707 10.1097/00043426-200102000-00014

[vms31402-bib-0029] Ragipoglu, D. , Dudeck, A. , Haffner‐Luntzer, M. , Voss, M. , Kroner, J. , Ignatius, A. , & Fischer, V. (2020). The role of mast cells in bone metabolism and bone disorders. Frontiers in Immunology, 11, 163. 32117297 10.3389/fimmu.2020.00163 PMC7025484

[vms31402-bib-0030] Raymond, L. M. , Funk, T. , Braziel, R. M. , Fan, G. , Gatter, K. , Loriaux, M. , Dunlap, J. , & Raess, P. W. (2020). Mast cell sarcoma with concurrent mast cell leukaemia. British Journal of Haematology, 189, e160–e164. 32242922 10.1111/bjh.16581

[vms31402-bib-0031] Sfiligoi, G. , Rassnick, K. M. , Scarlett, J. M. , Northrup, N. C. , & Gieger, T. L. (2006). Outcome of dogs with mast cell tumors in the inguinal or perineal region versus other cutaneous locations‐ 124 cases. Journal of the American Veterinary Medical Association, 226(8), 1368–1374. 10.2460/javma.2005.226.1368 15844431

[vms31402-bib-0032] Monnier, J. , Georgin‐Lavialle, S. , Canioni, D. , Lhermitte, L. , Soussan, M. , Arock, M. , Bruneau, J. , Dubreuil, P. , Bodemer, C. , Chandesris, M. O. , Lortholary, O. , Hermine, O. , & Damaj, G. (2016). Mast cell sarcoma – New cases and literature review.pdf. Oncotarget, 7(40), 66299–66309. 27602777 10.18632/oncotarget.11812 PMC5323235

[vms31402-bib-0033] Sledge, D. G. , Webster, J. , & Kiupel, M. (2016). Canine cutaneous mast cell tumors: A combined clinical and pathologic approach to diagnosis, prognosis, and treatment selection. Veterinary Journal, 215, 43–54. 27372911 10.1016/j.tvjl.2016.06.003

[vms31402-bib-0034] Solomou, A. G. (2017). Magnetic resonance imaging of pineal tumors and drop metastases: A review approach. Rare Tumors, 9, 6715. 29142658 10.4081/rt.2017.6715 PMC5661140

[vms31402-bib-0035] Stefanello, D. , Valenti, P. , Faverzani, S. , Bronzo, V. , Fiorbianco, V. , Pinto Da Cunha, N. , Romussi, S. , Cantatore, M. , & Caniatti, M. (2009). Ultrasound‐guided cytology of spleen and liver: A prognostic tool in canine cutaneous mast cell tumor. Journal of Veterinary Internal Medicine, 23, 1051–1057. 19656285 10.1111/j.1939-1676.2009.0354.x

[vms31402-bib-0036] Steinmetz, A. (2017). Superior orbitectomy and chemotherapy in a dog with frontal sinus squamous cell carcinoma: A case report and review of the literature. Clinical Case Reports, 5, 513–520. 28396780 10.1002/ccr3.889 PMC5378860

[vms31402-bib-0037] Terpos, E. , Ntanasis‐Stathopoulos, I. , Gavriatopoulou, M. , & Dimopoulos, M. A. (2018). Pathogenesis of bone disease in multiple myeloma: From bench to bedside. Blood Cancer Journal, 8, 7. 29330358 10.1038/s41408-017-0037-4 PMC5802524

[vms31402-bib-0038] Takahashi, T. , Kadosawa, T. , Nagase, M. , Matsunaga, S. , Mochizuki, M. , Nishimura, R. , & Sasaki, N. (2000). Visceral mast cell tumors in dogs: 10 cases (1982–1997). Journal of the American Veterinary Association, 216(2), 222–226. 10.2460/javma.2000.216.222 10649758

[vms31402-bib-0039] Vigeral, M. , Bentley, R. T. , Rancilio, N. J. , Miller, M. A. , & Heng, H. G. (2018). Imaging diagnosis ‐antemortem detection of oligodendroglioma “Cerebrospinal Fluid Drop Metastases” in a dog by serial magnetic resonance imaging. Veterinary Radiology & Ultrasound: The Official Journal of the American College of Veterinary Radiology and the International Veterinary Radiology Association, 59, E32–E37. 28176389 10.1111/vru.12474

[vms31402-bib-0040] Weishaar, K. M. , Thamm, D. H. , Worley, D. R. , & Kamstock, D. A. (2014). Correlation of nodal mast cells with clinical outcome in dogs with mast cell tumour and a proposed classification system for the evaluation of node metastasis. Journal of Comparative Pathology, 151, 329–338. 25172053 10.1016/j.jcpa.2014.07.004

[vms31402-bib-0041] Welle, M. M. , Bley, C. R. , Howard, J. , & Rüfenacht, S. (2008). Canine mast cell tumours: A review of the pathogenesis, clinical features, pathology and treatment. Veterinary Dermatology, 19, 321–339. 18980632 10.1111/j.1365-3164.2008.00694.x

[vms31402-bib-0042] Willmann, M. , Yuzbasiyan‐Gurkan, V. , Marconato, L. , Dacasto, M. , Hadzijusufovic, E. , Hermine, O. , Sadovnik, I. , Gamperl, S. , Schneeweiss‐Gleixner, M. , Gleixner, K. V. , Böhm, T. , Peter, B. , Eisenwort, G. , Moriggl, R. , Li, Z. , Jawhar, M. , Sotlar, K. , Jensen‐Jarolim, E. , Sexl, V. , …, Valent, P. (2021). Proposed diagnostic criteria and classification of canine mast cell neoplasms: A consensus proposal. Frontiers in Veterinary Science, 8, 755258. 34957277 10.3389/fvets.2021.755258 PMC8702826

[vms31402-bib-0043] Yang, N. S. , Griffin, L. R. , Frank, C. B. , & Bartner, L. R. (2022). What is your diagnosis? Journal of the American Veterinary Medical Association, 259, 1–4. 10.2460/javma.20.10.0592 35290198

